# Learning to smell danger: acquired associative representation of threat in the olfactory cortex

**DOI:** 10.3389/fnbeh.2014.00098

**Published:** 2014-04-07

**Authors:** Wen Li

**Affiliations:** ^1^Department of Psychology, University of Wisconsin-MadisonMadison, WI, USA; ^2^Waisman Center, University of Wisconsin-MadisonMadison, WI, USA

**Keywords:** threat encoding, olfactory sensory cortex, acquired associative representation, aversive conditioning, olfaction, anxiety

## Abstract

Neuroscience research over the past few decades has reached a strong consensus that the amygdala plays a key role in emotion processing. However, many questions remain unanswered, especially concerning emotion perception. Based on mnemonic theories of olfactory perception and in light of the highly associative nature of olfactory cortical processing, here I propose a sensory cortical model of olfactory threat perception (i.e., sensory-cortex-based threat perception): the olfactory cortex stores threat codes as acquired associative representations (AARs) formed via aversive life experiences, thereby enabling encoding of threat cues during sensory processing. Rodent and human research in olfactory aversive conditioning was reviewed, indicating learning-induced plasticity in the amygdala and the olfactory piriform cortex. In addition, as aversive learning becomes consolidated in the amygdala, the associative olfactory (piriform) cortex may undergo (long-term) plastic changes, resulting in modified neural response patterns that underpin threat AARs. This proposal thus brings forward a sensory cortical pathway to threat processing (in addition to amygdala-based processes), potentially accounting for an alternative mechanism underlying the pathophysiology of anxiety and depression.

## Introduction

Whether it is roaming on the safari or dozing by the fireplace, the ability to quickly detect threat (e.g., a tiger or a burning rug) and initiate appropriate responses can mean life or death for an organism. Decades of neuroscience research in emotion processing has reached a strong consensus: the amygdala extracts biological significance of a sensory cue and initiates and controls affective and motivational responses to the stimulus (LeDoux, 2000, 2012; Adolphs, 2013). In terms of threat perception, a widely held view is that the amygdala projects emotionally charged outputs to the sensory cortex, thereby enabling perceptual analysis of potential danger (Phelps and LeDoux, 2005; Vuilleumier and Pourtois, 2007). These theories have been incorporated into neural models of emotional disorders, shedding important light on the pathophysiology of anxiety and depression (Davis, 1992; Rauch et al., 2006; Clark and Beck, 2010).

However, striking findings have arisen recently, suggesting that threat processing can operate independently of the amygdala. Patient S.M. an individual with complete bilateral amygdala lesions, demonstrated intact early threat perception (Tsuchiya et al., 2009); she and two other patients with similar amygdala lesions also developed panic attacks and intense fear when challenged with high concentration CO_2_ (Feinstein et al., 2013). In addition, extensive research indicates very swift (at latencies around 100 ms) threat processing in the visual cortex (Li et al., 2007, 2008b; Krusemark and Li, 2011, 2013; Forscher and Li, 2012; also cf. Vuilleumier and Pourtois, 2007), preceding the latencies of threat processing measured with depth-electrode recording in the amygdala (Oya et al., 2002; Krolak-Salmon et al., 2004). Accordingly, instead of relying on amygdala input, early threat perception may depend on inputs from other brain areas (e.g., the orbitofrontal cortex; Barrett and Bar, 2009), or simply consummate during the initial sensory feedforward sweep. Indeed, a seminal paper by Pessoa and Adolphs (2010) promotes a multi-path framework, arguing for extra-amygdala neural circuits in threat processing.

Towards that end, this article draws evidence from long-standing animal research and recent human data (reviewed below), proposing a sensory cortical model of threat perception (i.e., sensory-cortex-based threat perception): the sensory cortex stores threat codes/representations, thereby enabling perceptual encoding of threat information once an environmental input reaches the sensory cortex. This model highlights an *active*, *independent* role of the sensory cortex in threat perception, as opposed to the conventionally understood role of passively processing/integrating threat-laden inputs generated elsewhere in the brain (e.g., the amygdala; Phelps and LeDoux, 2005; Vuilleumier and Pourtois, 2007). This model makes clear evolutionary sense by permitting categorization of biological significance in the stage of sensory analysis, prompting an organism to respond with minimal delay. Importantly, by putting forward a sensory-based mechanism, in addition to limbic-based threat processing, this model would help reconcile controversial findings in the literature as discussed above, such as the very swift threat perception, even in the absence of intact amygdala.

## A (olfactory) sensory cortical model of threat perception

To provide a mechanistic explication of this account, a few principles need to be emphasized. As James (1890) asserted, “every perception is an acquired perception,” human perception is largely learned and depends on long-term memory (Goldstone, 1998; Stevenson and Boakes, 2003). This proposed model thus takes a learning perspective, building on mnemonically-based threat codes/representations acquired through life experiences. Indeed, except for a limited set of innate phobic objects (e.g., snakes; Ohman and Mineka, 2001), the copious repertoire of threat cues in humans appears to be learned and accumulated over the course of life, varying from the concrete (e.g., germs and guns) to the abstract (e.g., disease and death).

In addition, given the associative nature of memory and threat processing, this model centers on the associative (secondary) sensory cortex, characterized by dense intrinsic and extrinsic neural connections, as a primary site of threat code storage and threat encoding. In particular, extrinsic top-down connections transmitting contextual information can modulate sensory cortical activity to facilitate context-relevant behavior (Cohen and Maunsell, 2011; Harris and Mrsic-Flogel, 2013); this focus on associative sensory cortex would thus permit context- or state-dependent flexibility (adaptive to affective, motivational and physiological states) in perceiving threat while ensuring sensory fidelity (Proffitt, 2006; Barsalou, 2008; Krusemark and Li, 2013; Krusemark et al., 2013).

Furthermore, I chose olfaction as a model system for this account. The olfactory cortex has served as a model system for the cortical representation of associative memory (Gluck and Granger, 1993; Haberly, 1998), owing to the fact that olfactory perception is deeply rooted in memory (Stevenson and Boakes, 2003; Wilson and Stevenson, 2003) and that olfactory cortical processing is highly associative (Wilson and Sullivan, 2011). Moreover, akin to threat encoding specifically, olfaction is uniquely related to emotion in function and anatomy (Schiffman, 1974; Carmichael et al., 1994), given their phylogenetic proximity. Studies have shown that olfactory perception shifts readily with a perceiver’s affective state (Herz et al., 2004; Chen and Dalton, 2005; Herz, 2005; Pollatos et al., 2007; Krusemark et al., 2013); odor affective value (vs. odor character, “lemon” or “orange”) may even represent the dominant dimension in olfactory perception (Yeshurun and Sobel, 2010). Finally, odor hedonicity is posited to be borne directly out of emotional experiences attached to an odor (Herz, 2005). Taken together, these properties of olfactory perception represent a particularly close fit to the model here.

Mechanistically, this proposed account rests on long-term plasticity (as a form of long-term memory storage) in the sensory cortex, consequent to aversive associative learning. As accruing evidence suggests that the sensory cortex contains richly interconnected neurons, whose patterns of firing as a whole encode sensory input (Harris and Mrsic-Flogel, 2013), this model highlights modified neural response patterns induced by long-term plasticity in the sensory cortex. Critically, these patterns would reflect acquired associative representations (AARs) that encode the threat meanings learned via negative experiences (in addition to the sensory features of the stimuli). As such, constituting sensory neural codes of acquired threats, these threat AARs would underpin threat perception in the sensory cortex.

**Figure 1:**
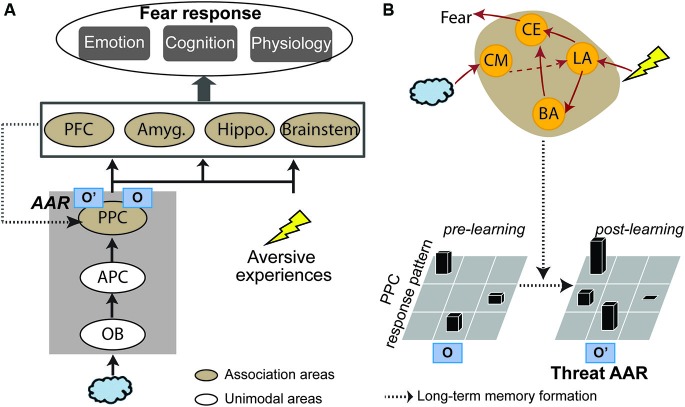
**A sensory cortical model of olfactory threat encoding**. **(A)** When an odor is co-experienced with an aversive event, associative learning may occur. Long-term plasticity induced by such learning results in long-lasting changes in the olfactory (posterior piriform) cortical response pattern to the CS odor. As such, original representation of the odor (O) turns into acquired associative representation/AAR (O’). Such threat AARs constitute the basis of sensory cortical encoding of threat. Later encounters of the same odor will activate O’ to directly support threat encoding and trigger emotion responding. **(B)** Neural mechanisms. Initial association between the odor and aversive experience is formed in the lateral amygdala (LA), which projects directly or indirectly (via the basal nucleus of amygdala/BA) to the central nucleus (CE) to initiate and control fear responses. Over time, the acquired association is converted into a long-term memory stored in the PPC in the form of a threat AAR. Possible mediating mechanisms are increases in amygdala theta oscillation, cholinergic activity and amygdala efferents to the PPC. APC = anterior piriform cortex; OB = olfactory bulb; PFC = prefrontal cortex; Amyg. = amygdala; Hippo = hippocampus; CM = corticomedial nucleus of amygdala.

Based on the fear learning literature reviewed below, the genesis of threat-relevant sensory cortical long-term plasticity and threat AARs could involve two components (Figure 1): (1) acquisition/consolidation of aversive associative learning in the amygdala, thereby attaching threat meanings to innocuous odors; and (2) over time, the initial amygdala-based learning gives rise to long-term sensory cortical plasticity. That is, the associative olfactory (posterior piriform) cortex (PPC) undergoes plastic changes, resulting in an updated neural response pattern (i.e., a threat AAR) to the conditioned odor. Accordingly, subsequent encounters of the conditioned odor will activate this threat AAR in the PPC, supporting olfactory cortical encoding of threat. Finally, outputs from this sensory process (i.e., threat-laden sensory impulses) can trigger fear responses via projections to a wide range of associative neural networks (especially the amygdala, prefrontal cortex and brain stem structures).

## Amygdala mediates the acquisition/consolidation of olfactory aversive conditioning

It is a well-known fact that repeated paired stimulation of a stimulus (CS, e.g., a tone) and a salient stimulus (US, e.g., an electric shock or a drop of water) often result in conditioning (e.g., Pavlovian or emotional conditioning), and the CS thus acquires a new threat/reward meaning (Pavlov, 1927/1960). In terms of the neural mechanism, extensive research has ascribed a key role to the amygdala, especially the basolateral complex (comprising the lateral, basal and accessory basal nuclei), in aversive conditioning. As described in influential reviews (LeDoux, 2000, 2012; Maren and Quirk, 2004; Myers and Davis, 2007), the lateral nucleus of amygdala reliably exhibits increased spike firing and long-term potentiation during conditioning, underscoring the lateral nucleus as a primary site of conditioning acquisition and consolidation. Furthermore, pre-training damage to the lateral nucleus directly impairs fear conditioning, indicative of its causal role in this process. Via direct or indirect intra-amygdala connections, the lateral nucleus triggers activation of the central nucleus of the amygdala, which initiates and controls the expression of the acquired fear via projections to a set of midbrain and brainstem structures (e.g., hypothalamus and periaqueductal gray). Finally, it is worth noting that for auditory fear conditioning, the magnocellular medial geniculate nucleus could mediate the initial learning (Weinberger, 2011).

The aversive conditioning literature has primarily involved the auditory sense and, to a lesser extent, the visual sense. Nevertheless, olfactory conditioning research has yielded similar conclusions (cf. Mouly and Sullivan, 2010). Electrophysiological studies in rodents indicate that the basolateral amygdala exhibits potentiated responses during olfactory aversive conditioning (Rosenkranz and Grace, 2002) and shortly after (Rattiner et al., 2004; Sevelinges et al., 2004). Highlighting its critical role in olfactory conditioning acquisition, pre-training lesions or pharmacological inactivation/inhibition of the basolateral amygdala significantly reduces conditioned fear or aversion to the CS odor (Cousens and Otto, 1998; Wallace and Rosen, 2001; Walker et al., 2005; Miranda et al., 2007; Sevelinges et al., 2009). Furthermore, post-training inactivation of the basolateral amygdala (Kilpatrick and Cahill, 2003; Sevelinges et al., 2009) would largely attenuate conditioned aversion, implicating this area in the consolidation of olfactory aversive associative learning. Using biomarkers of synaptic plasticity to reflect fear learning, research also further reveals plastic changes, during or shortly after conditioning, in the basolateral amygdala of rodents exposed to paired odor-shock stimulation, in the form of heightened expression of brain derived neurotrophic factor (BDNF; Jones et al., 2007) or increased concentrations of glutamate and GABA (Hegoburu et al., 2009).

**Figure 2:**
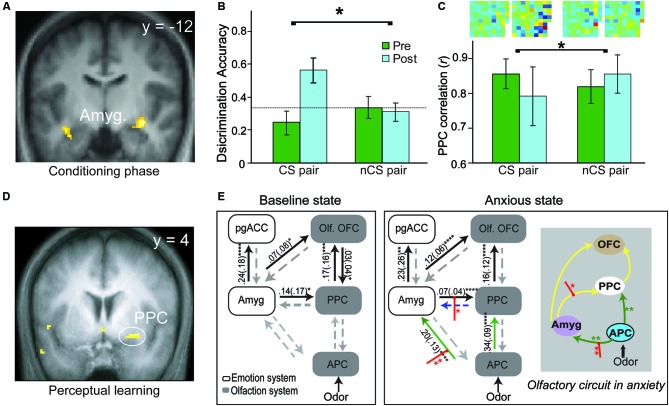
**Olfactory aversive learning and neural circuitry adaptation in humans. (A)** During the conditioning phase, the amygdala exhibits conditioned response to the conditioned odor and its extremely similar enantiomer counterpart. **(B)** Perceptual discrimination between the CS and its enantiomer counterpart improves markedly after conditioning whereas the unconditioned enantiomer pair remains indistinguishable. **(C)** In parallel, response patterns for the CS pair become divergent (relative to the non-conditioned/nCS pair). Differential odor maps (spatial configurations of response intensities in all active PPC voxels) within each pair are displayed at the top of the bar graph, with strong-colored voxels reflecting large disparities between the counterparts. Notably, the post-conditioning differential map for the CS pair contains far more voxels of strong colors. **(D)** Plasticity in the PPC—enhanced response to the target odor after prolonged mere exposure. **(E)** The olfactory sensory pathway adapts readily with induced anxiety, characterized by strengthened APC efforts to amygdala and PPC, and amplified amygdala efferent to the PPC. This olfactory circuitry reorganization is accompanied by a significant negative shift in perceived pleasantness of odors (not shown here). This enhanced amygdala-olfactory-cortex connection may facilitate the transfer of learning from the amygdala to the olfactory cortex. Yellow lines represent intrinsic connections initially significant, green lines those that become significant in anxiety and red intercepting lines modulation by odors in anxiety. OFC = orbitofrontal cortex; PPC = posterior piriform cortex; pgACC = pregenual anterior cingulate cortex; Amyg. = amygdala; olf. = olfactory; APC = anterior piriform cortex. Panels A–C are adapted from Li et al. (2008a), Panel D from Li et al. (2006) and Panel E from Krusemark et al. (2013).

A substantial body of human neuroimaging research in aversive conditioning has emerged, albeit largely concerning auditory and visual CS (Sehlmeyer et al., 2009). Human neuroimaging research of olfactory aversive conditioning remains scant. Functional magnetic resonance imaging (fMRI) data from our lab indicate conditioned responses evoked by the CS odor during the acquisition phase (Figure 2A, Li et al., 2008a). That is, conforming to an exponential decay observed in prior imaging studies of visual aversive conditioning (Büchel et al., 1998; LaBar et al., 1998), the amygdala response to the CS odor (vs. CS- odor) increases sharply in early trials and declines in later trials. In addition, pairing odors with painful (CO_2_) trigeminal stimulation in human subjects, a new fMRI study reveals significant response enhancement to the CS odor in the amygdala during conditioning (Moessnang et al., 2013). These extant findings thus concur with conclusions of the general literature, confirming the role of amygdala in the acquisition of human olfactory conditioning.

Notably, the olfactory anatomy is fairly distinct from other senses; it lacks the thalamic relay critical for signal transmission in other modalities, and its inputs terminate in the corticomedial nucleus of amygdala (Carmichael et al., 1994) versus the lateral nucleus for other sensory inputs (Luskin and Price, 1983; Savander et al., 1996). Despite these disparities, fear learning in the amygdala is fairly generic, contrasting with sensory-specific plasticity in the olfactory cortex.

## Olfactory cortex supports acquired associative representations (AARs) via olfactory aversive conditioning

### Transfer of aversive associative learning from the amygdala to the olfactory cortex

As discussed earlier, the current model requires long-term learning-based plasticity in the sensory cortex to substantiate threat codes (threat AARs). In fact, although the role of amygdala in long-term fear memory is still debatable (LeDoux, 2000; McGaugh, 2004), the sensory cortex has long been implicated as a site of storage and retrieval of remote associative memory (Mishkin, 1982; Squire, 1987; Damasio, 1989). Namely, long-standing views posit that as the memory of an object becomes fully consolidated in mediotemporal structures (e.g., the hippocampus), the object-specific sensory cortex (e.g., auditory cortex to a tone or olfactory cortex to an odor) gradually takes over to support long-term storage of the memory (Haberly and Bower, 1989; Gluck and Granger, 1993; Gluck and Myers, 1993; Squire and Wixted, 2011). Regarding aversive associative learning in particular, the sensory cortex could undergo plastic changes to serve as a primary storage site of long-term fear memory, following the initial fear learning (Weinberger, 2004).

Three mechanisms may mediate this transfer (Figure 1B). Firstly, long-range, low-frequency (theta) oscillatory activity in the amygdala is potentiated following conditioning, thereby facilitating amygdala interaction with sensory cortical storage sites to induce plasticity in these regions (Haberly and Bower, 1989; Gluck and Granger, 1993; Paré et al., 2002). Secondly, aversive conditioning potentiates amygdala efferents to the nucleus basalis, driving its acetylcholine release in the sensory cortex to mediate long-lasting sensory cortical plasticity (McGaugh et al., 2002; Weinberger, 2007). Thirdly, negative affective states induced by conditioning can intensify amygdala efferents to the sensory cortex to induce cortical plasticity, as suggested by a recent human fMRI in our lab (Krusemark et al., 2013). Combining dynamic causal connectivity analysis (Friston et al., 2013) and anxiety induction in a simple odor detection task, we demonstrate that an induced anxious state can reorganize the olfactory sensory circuitry, incorporating the amygdala as an integral step. That is, following anxiety induction, initially insignificant efferents from the anterior piriform cortex (APC) to the amygdala become important; also, efferents from the amygdala to PPC are further strengthened (Figure 2E). Notably, this circuitry reorganization is accompanied by a negative shift in perceived odor valence. Conceivably, by (almost invariably) inducing anxious/negative affective states, aversive conditioning can similarly enhance amygdala discharges to the PPC, promoting plastic changes in this area.

In consequence, long-lasting plasticity would arise in the sensory cortex, which then selectively updates neuronal ensemble response patterns to the CS, substantiating the long-term memory of acquired threat value in the CS. Notably, Weinberger’s lab was the first to show that the primary auditory cortex is the locus of long-term plasticity due to auditory fear conditioning, supporting altered sensory encoding of auditory CS (Weinberger, 2007, 2011; Weinberger and Bieszczad, 2011). To date, there has been considerable evidence of olfactory cortical plasticity as a result of olfactory aversive conditioning.

### Olfactory cortical plasticity induced by olfactory aversive conditioning

The olfactory cortex consists of the anterior olfactory nucleus, olfactory tubercle, cortical nucleus of the amygdala, piriform cortex and entorhinal cortex (Carmichael et al., 1994; Shipley and Ennis, 1996; Haberly, 1998). The piriform cortex, divided into anterior and posterior piriform cortices (APC and PPC), is the largest subarea of the olfactory cortex. As described in excellent recent reviews (Gottfried, 2010; Mori and Sakano, 2011; Wilson and Sullivan, 2011), the APC serves as a primary olfactory cortex influenced strongly by bulbar mitral cell afferents and thus maintains considerable fidelity to the molecular properties of an odorant, whereas the PPC anatomically and functionally resembles a higher-level association cortex, supporting higher-order olfactory perception (e.g., odor quality encoding and categorization). Therefore, the PPC is postulated as the primary locus of olfactory threat AARs and threat encoding in the current model (Figure 1).

Indeed, akin to the associative and malleable nature of PPC, computational modeling and *in vitro* physiological studies suggest that long-term potentiation is more readily induced in the PPC (than APC), enabling long-term memory storage, whereas the APC is more associated with sensory processing and simple forms of short-term memory (Lynch and Granger, 1989; Jung et al., 1990). Empirical data of olfactory aversive conditioning largely concur with this view. After olfactory conditioning, PPC (but not APC) in the trained animals exhibits stronger local field potentials (Litaudon et al., 1997; Sevelinges et al., 2004) or BDNF expression (Jones et al., 2007) compared to the baseline. Hegoburu et al. (2009) also demonstrate increases in GABA and glutamate concentrations in the PPC of trained rats. To note, this PPC plasticity persists up to 30 min into odor-shock conditioning, contrasting with transient concentration increases of these amino acids in the basolateral amygdala (observed in the same study). These distinct plasticity time courses may correspond to the differential functions of these two areas: the amygdala is critical for initial learning while the PPC is important for long-term memory of learning. It is also worth noting that CS-odor evoked response potentiation has been observed in the APC of awake rats using single-unit recording (Barnes et al., 2011; Chen et al., 2011). This positive finding could be ascribed to the efficacy of the methodology, but as PPC activity was not assessed in these studies, it is unclear whether PPC plasticity coexisted or even mediated the APC changes. Finally, recent evidence also suggests that fear learning can induce plasticity in even lower levels of the olfactory hierarchy (i.e., olfactory receptor neurons and the olfactory bulb; Jones et al., 2008; Kass et al., 2013).

Concerning human evidence, our aforementioned conditioning study also shows that the PPC (but not APC) exhibits significant CS-evoked response changes, paralleling the rodent findings (Figure 2C, Li et al., 2008a); this superior plasticity in PPC further accords with a previous fMRI study in the lab involving simple perceptual learning, where the PPC but not APC demonstrates response enhancement following prolonged odor exposure (Li et al., 2006; Figure 2D). Critically, our conditioning study reveals that behavioral and PPC neuronal ensemble responses to two initially indistinguishable odor enantiomers (mirror-image molecules) become distinct after one of them is paired with electric shock (Figures 2B and **C**, Li et al., 2008a). This divergence contrasts with convergent response augmentation in the amygdala to both the CS odor and its non-conditioned enantiomer counterpart (Figure 2A). These findings thus emphasize that PPC plasticity is selective to the CS odor, independent of amygdala plasticity that is generalized to similar cues. This specialized PPC plasticity could thus underpin sensory cortical representation of the acquired threat value in the CS odor, supporting discrimination perception of the CS odor versus its counterpart following conditioning.

Finally, direct evidence for *long-term storage* of aversive conditioning in the olfactory cortex has emerged from a series of experiments conducted by Sacco and Sacchetti (2010). The authors demonstrate long-term memory of shock conditioning: trained rats exhibit strong freezing responses to the CS one month post-training. Nevertheless, lesioning CS-specific secondary sensory (visual, auditory and olfactory) cortex (including the PPC), one month post-training, largely abolishes the conditioned responses. Notably, lesions to the secondary sensory cortex do not impair new fear learning or recent (e.g., 1 day post-training) fear memories; this coincides with previous research which induced sensory cortical lesions either before or shortly after conditioning and failed to find impairment in fearing learning or recent fear memory (Romanski and LeDoux, 1992; Rosen et al., 1992; Falls and Davis, 1993; Campeau and Davis, 1995), suggesting that the sensory cortex is not critical for acquisition and consolidation of fear memory. Nonetheless, this recent study evinces that the secondary sensory cortex is essential for long-term storage and retrieval of acquired threat value in the CS. That is, as time elapses after initial learning, the CS will need to activate the threat AAR stored in the secondary (associative) sensory cortex to elicit conditioned responses.

## Conclusion

This review integrates mnemonic theories of olfaction and evidence of olfactory aversive associative learning, promoting a sensory cortical model of threat perception. The amygdala may mediate olfactory associative learning and transfer this learning to the olfactory cortex. The consequent long-term plasticity in the olfactory associative cortex (PPC) may serve to support olfactory threat AARs (representing acquired threat value in conditioned odors). These threat AARs can independently enable sensory cortical encoding of threat and trigger various responses via projections to associative neural networks. This sensory pathway may specialize in mandatory, reflexive, and sensory-specific forms of threat encoding, whereas the amygdala, especially via interaction with sensory and ventral prefrontal cortices, may chiefly be responsible for flexible, context-relevant, and amodal (abstract) threat processing (Krusemark and Li, 2013). In addition, these parallel systems may confer further ecological advantage by integrating a fine-tuned sensory module for *specific* threat identification and a broadly-tuned amygdala module for *sensitive* threat detection (Li et al., 2008a). By elucidating threat encoding in the sensory cortex, this proposed model may provide new insights into the pathophysiology of emotional disorders, pointing to a concrete clinical intervention target in the sensory cortex.

## Limitations and future directions

Compared to rich data in auditory fear learning (Weinberger, 2004, 2007), a limitation of the olfactory fear learning literature is the limited evidence of long-term plasticity/memory storage in the sensory cortex, which is a critical neural basis of the neurosensory account of threat perception. That is, very few studies in this literature have assessed olfactory sensory cortical plasticity after a prolonged delay (e.g., 2 weeks or 1 month after initial learning), and there is virtually no human evidence. Therefore, future research is warranted to isolate evidence of long-term memory of acquired threat in the sensory cortex, especially in human subjects.

Another notable limitation concerns the spatial resolution of the fMRI methodology, a first-line non-invasive method in probing neural activities in humans. With millions of neurons in each fMRI voxel (a volume of few cube millimeters; Logothetis, 2008), patterns of voxel-wise fMRI signal intensity would reflect large-scale configurations of neural ensemble activity as opposed to single-unit neuronal response patterns. Also, various methods have been applied in pattern-based fMRI analysis, warranting comparisons and cross-validations of those findings in the future. That said, it is also worth noting that given the considerable level of redundant coding in neuronal populations in the sensory cortex (e.g., the often correlated firing of neighboring neurons; Smith and Kohn, 2008; Luczak et al., 2009), these large-scale patterns observed in humans could still provide useful insights into sensory coding, especially when integrated with animal electrophysiological data.

## Conflict of interest statement

The author declares that the research was conducted in the absence of any commercial or financial relationships that could be construed as a potential conflict of interest.
